# The impact of flexible human resource management on employees’ workplace well-being based on self-determination theory

**DOI:** 10.3389/fpsyg.2026.1885266

**Published:** 2026-08-04

**Authors:** Yixin Zhang, Mengli Liu, Tianyu Du, Weipeng Lin, Xiaohui Qu, Bing Liu

**Affiliations:** 1 School of Management, Shandong University, Jinan, China; 2 Xuzhou College of Industrial Technology, Xuzhou, China; 3 State-owned Assets Supervision and Administration Bureau of Taishan District, Taian, China

**Keywords:** basic psychological needs, flexible human resource management, HRM system strength, self-determination theory, workplace well-being

## Abstract

**Introduction:**

Flexible human resource management (FHRM) has become an effective means for enhancing an organization’s adaptability and responsiveness through the efficient acquisition and optimal deployment of human resources. Although previous studies argued that FHRM can positively influence organizational and individual overt behavioral outcomes, some overlooked its impact on the well-being of employees.

**Methods:**

By drawing on self-determination theory, we propose that FHRM can satisfy the basic psychological needs of employees and thus enhance their workplace well-being. Moreover, we posit that the high HRM system strength can enable the management to reach a consensus and foster the creation of a positive climate for FHRM content and thus satisfy the basic psychological needs of employees. We test our hypotheses by conducting a two-phase survey. We collect 81 paired questionnaires from 81 leaders and 444 employees.

**Results:**

Our results confirmed that FHRM was positively associated with employees’ workplace well-being. Satisfaction of basic psychological needs (autonomy, competence, and relatedness) mediated this relationship. In addition, HRM system strength moderated the relationship between FHRM and satisfaction of need for relatedness. Specifically, the indirect effect of FHRM on workplace well-being via satisfaction of need for relatedness was stronger when HRM system strength was high than when it was low.

**Discussion:**

Our research can deepen the understanding of FHRM by revealing its impact on the satisfaction of the basic psychological needs and employees’ workplace well-being.

## Introduction

The internal and external environments of enterprises are changing rapidly in this era of volatility, uncertainty, complexity, and ambiguity (VUCA), which has placed high demand on ability to adapt to changes. To address the impact of environmental uncertainty, an increasing number of enterprises have implemented flexible human resource management (FHRM) to enhance their organizational adaptability and responsiveness (Chang et al., 2013). FHRM can be defined as a set of consistent human resource practices that can facilitate the acquisition and development, as well as the rapid and effective redeployment, of human resources (Bal and De Lange, 2015; Chang et al., 2013). Owing to its adaptability in effectively addressing challenges in this VUCA era, FHRM and its outcomes have attracted increasing attention from scholars.

Prior research on FHRM has primarily focused on its organizational-level consequences, including firm capabilities, innovation, and performance. Specifically, FHRM enhances absorptive capacity (Ben Guedria et al., 2025; Chang et al., 2013), organizational resilience (Li and Lin, 2024), and intellectual capital (Lakshman et al., 2022), while also promoting innovation (Chang et al., 2013; Lakshman et al., 2022; Sangadji and Islami, 2024; Whyman and Petrescu, 2013) and organizational ambidexterity (Islam et al., 2025). Ultimately, FHRM contributes to overall firm performance (Pahi et al., 2024), sustainable performance (Islam et al., 2026), and market responsiveness (Chang et al., 2013). Given that employees are the ultimate recipients of HR practices, recent research has also examined the individual-level effects of FHRM. Most studies have reported positive outcomes, including enhanced work engagement, organizational citizenship behavior, innovative behavior, and task performance, which in turn reduce work–family conflict and turnover intentions (Allen et al., 2013; Bal and De Lange, 2015; Lee et al., 2011; Zhang et al., 2023). Recently, however, scholars have also identified a potential dark side, suggesting that FHRM may encourage prosocial rule breaking (Chen and Hu, 2026).

Notably, HRM should strike a balance between the dual objectives of organizational performance and employees’ workplace well-being to ensure that the employees and the organization benefit (Brown et al., 2008; Guest, 2017). Meanwhile, enhancement of employees’ workplace well-being has become an important source of competitive advantage for organizations (Koys, 2001). It also can help organizations effectively predict their employees’ emotional commitment and job performance (Wright and Cropanzano, 2004). However, current research on FHRM focuses on its impact on employees’ overt behavioral outcomes but neglects its effect on psychological dimensions, such as employees’ workplace well-being. Therefore, our research aims to explore the relationship between FHRM and employees’ workplace well-being.

Self-determination theory can provide a theoretical framework for understanding how individual psychological situations can influence workplace well-being (Deci et al., 2017). The theory proposes that employees can attain a positive psychological state in the workplace if their working environment meets their basic psychological needs, including autonomy, competence, and relatedness (Deci et al., 2001). Therefore, to understand the relationship between FHRM and employees’ workplace well-being, we posit that FHRM can satisfy employees’ basic psychological needs and thus enhance their workplace well-being.

In terms of the impact of HRM, scholars have emphasized the importance of its efficient implementation, in addition to practice (Bowen and Ostroff, 2004). Other scholars found that HRM system strength, which refers to the effectiveness of such system in conveying the different types of information needed to create a strong situation, is an important factor for understanding the efficient implementation of HRM (Bowen and Ostroff, 2004). Unlike HRM practices, which have specific and practicable goals grounded in practice, HRM system strength is a connecting mechanism that can establish collective shared beliefs, attitudes, and behaviors among employees (Bowen and Ostroff, 2004). Thus, organizations with a high HRM system strength can deliver HRM information efficiently to their employees and contribute to the creation of a positive psychological climate in the workplace. Therefore, we consider HRM system strength as a moderator in the relationship between FHRM and the satisfaction of employees’ basic psychological needs.

**Figure 1:**
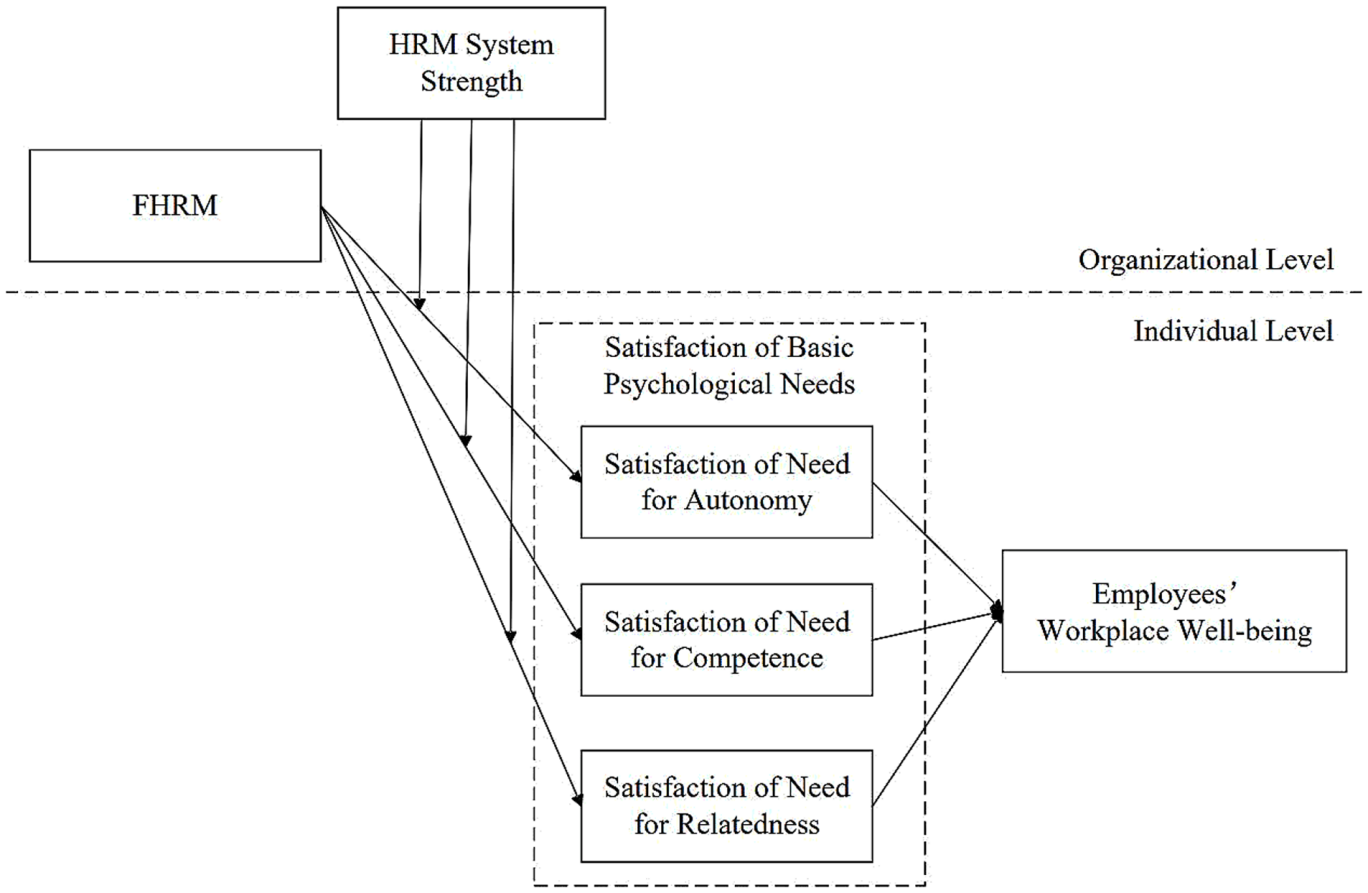
Theoretical model.

Our study makes three contributions to the literature. First, our research expands research on the psychological impact of FHRM on employees. Existing theories posit that FHRM can exert a positive impact on organizational and individual overt behavioral outcomes (Allen et al., 2013; Bal and De Lange, 2015; Chang et al., 2013; Lee et al., 2011; Sánchez et al., 2007; Whyman and Petrescu, 2013) but overlook its effect on individuals’ psychological dimensions. Our study extends research on FHRM outcomes at the individual level. Second, our study enriches research on the relationship between FHRM and employees’ well-being and thus extends the application of self-determination theory in the relationship between HRM practices and employees’ psychological experiences. Third, our study expands the boundary mechanisms of FHRM and deepens our understanding of the effect of FHRM practices by emphasizing the interaction of HRM practices and HRM system strength (see Figure 1).

## Theory and hypothesis development

### FHRM and employees’ workplace well-being

As an important organizational environment factor, HRM practices can be regarded as a channel through which organizations provide resources and support to their employees to help them deal with pressure (Boon and Kalshoven, 2014). FHRM refers to a series of internally consistent HRM practices that can be used to deploy employees quickly and efficiently (Bal and De Lange, 2015; Chang et al., 2013). Resource flexibility and coordination flexibility have been proven to be FHRM characteristics that can enhance employees’ workplace well-being (Chang et al., 2013).

Specifically, resource flexibility-oriented HRM involves the acquisition and development of human resources for a wide range of alternative uses (Chang et al., 2013). HRM practices can broaden employees’ knowledge base through general and extensive training, job rotation, and broadly designed jobs (Fried and Ferris, 1987). HRM practices can enable employees to meet work demands and attain their goals and thus enhance their workplace well-being (Huang et al., 2016). Meanwhile, coordination flexibility-oriented HRM involves the rapid and effective redeployment of human resources and related HRM practices, such as participative management, to motivate employees to accept transitions and redefine individual tasks, if necessary (Allen et al., 2013; Chang et al., 2013; Jeffrey Hill et al., 2008). Hence, HRM practices can enable employees to not only identify and utilize development opportunities effectively but also perceive organization-recognized growth and thus improve their workplace well-being (Kuvaas, 2008).

Therefore, in this study, we propose the following hypothesis:

*H1:* FHRM can positively affect employees’ workplace well-being.

### Impact of FHRM on employees’ workplace well-being through satisfaction of basic psychological needs

Self-determination theory posits that individuals have an inherent tendency to engage in self-development and seek internal growth, which require continuous encouragement from the external environment. When the external environment can satisfy individuals’ three basic psychological needs, namely, autonomy, competence, and relatedness, it can enable individuals to attain a positive psychological state, which in turn can influence their well-being, job satisfaction, organizational commitment, work engagement, and work performance (Baard et al., 2004; Deci et al., 2001; Gagné and Deci, 2005; Rasskazova et al., 2016). As an important organizational situation factor, FHRM focuses on individual motivation and development and can support employees by meeting their basic psychological needs (Cheewakoset et al., 2023; Sangadji and Islami, 2024).

First, autonomy refers to an individual’s desire to make independent choices according to their will when engaging in various activities, feel unrestricted, and have the power to make decisions and control their actions (Ryan and Deci, 2000). FHRM emphasizes employee involvement and empowers employees by giving them numerous opportunities to participate in organizational decision making by sharing information and participating in corporate management (Chang et al., 2013). At the same time, organizations can act on employees’ suggestions and feedback to give them a sense of autonomy in their work (Chang et al., 2013). Moreover, by giving employees flexible work hours, locations, and methods, organizations will enable them to decide how to allocate their time and energy and thus control their work (Crawford et al., 2010; Sánchez et al., 2007). Hence, FHRM can satisfy employees’ need for autonomy by involving them in decision making and allowing them to plan their work independently. By drawing on self-determination theory, we posit that the satisfaction of employees’ need for autonomy will improve their workplace well-being (Conway et al., 2015; Deci et al., 2017; Gillet et al., 2012; Simões and Alarcão, 2014).

Second, competence refers to an individual’s ability to interact effectively with their social environment and complete work tasks or perception of being competent and in control of their work (Deci and Ryan, 2000). FHRM focuses on broadening employees’ knowledge and honing their skills by offering them training activities based on the dynamism of the external environment and opportunities to take on different roles and tasks (Chang et al., 2013; Vansteenkiste et al., 2020; Xu et al., 2023). FHRM also involves providing employees with training, job rotation options, and communication regimes that can equip them with the knowledge and skills necessary to meet their job requirements and effectively address the various challenges at work. Thus, FHRM can meet employees’ need for competence to overcome work challenges. Moreover, self-determination theory suggests that FHRM support can satisfy employees’ need for competence, which can lead to workplace well-being (Deci et al., 2017; Gillet et al., 2012; Simões and Alarcão, 2014).

Last, relatedness refers to an individual’s ability to perceive emotions such as care and love from others within their environment and themselves as being a valued member of their organization (Ryan and Deci, 2000). FHRM can enhance employees’ perception of their relationships within their teams and sense of belonging. In terms of relationships with team members, FHRM can promote communication, knowledge sharing, and collaboration through team performance evaluations and compensation management (Van den Broeck et al., 2016; Xu et al., 2023). With regard to relationships with leaders, FHRM can foster positive interpersonal relationships by empowering employees and providing them with timely feedback, which can make them feel recognized and supported by their leaders (Pahi et al., 2024). Therefore, FHRM can satisfy employees’ need for relatedness by facilitating their interaction with other members of their organization (Van den Broeck et al., 2010). By drawing on self-determination theory, we posit that a positive organization climate can satisfy employees’ need for relatedness, which in turn can improve their workplace well-being (Deci et al., 2017; Gillet et al., 2012; Simões and Alarcão, 2014).

Therefore, we propose the following hypotheses:

*H2a:* FHRM will have a positive indirect effect on employees’ workplace well-being through the satisfaction of their basic psychological need for autonomy.

*H2b:* FHRM will have a positive indirect effect on employees’ workplace well-being through the satisfaction of their basic psychological need for competence.

*H2c:* FHRM will have a positive indirect effect on employees’ workplace well-being through the satisfaction of their basic psychological need for relatedness.

### Moderating effect of HRM system strength

A HRM system adopted by an organization is generally an organic combination of HRM content and processes (Bowen and Ostroff, 2004). In other words, HRM relies on not only the rationality of related practices but also their implementation effectiveness, such as HRM system strength (Bowen and Ostroff, 2004; Delmotte et al., 2012). Specifically, from the perspective of content, HRM practices can be viewed as having a symbolic or signaling function, that is, they send organizational management messages to employees. However, from the perspective of processes, HRM practices may be interpreted idiosyncratically (Bowen and Ostroff, 2004; Guzzo and Noonan, 1994). Interpretation differentiation will negatively impact HRM results, despite the inherit benefits of the adopted practices. Accordingly, such practices can be implemented and used to attain certain goals only if they are understood, internalized, and performed by all the employees (Hauff et al., 2017).

A high HRM system strength can promote the development of shared perceptions and the creation of a positive organizational climate for HRM content to enable all the members of an organization to understand its HRM practices the same way (Bowen and Ostroff, 2004; Mischel, 1973). In an organization where HRM system strength is high, event-effect information on HRM practices will be transparent and accurate and disclosed consistently to every employee, regardless of their identity, the time, and the place (Bowen and Ostroff, 2004). At the same time, a high HRM system strength can send clear signals and enable employees to meet behavioral expectations by improving communication quality (Bowen and Ostroff, 2004; Hauff et al., 2017). Therefore, when an organization that implements FHRM has a high HRM system strength, all its employees will likely receive adequate information about the FHRM practices; reach a consensus about the purpose of the FHRM practices, such as encouraging participative management, offering training, and promoting information communication; and consistently behave as expected by the organization. Accordingly, their need for autonomy, competence, and relatedness will be satisfied by FHRM. However, in an organization where HRM system strength is low, FHRM information may be interpreted and internalized in different ways, which in turn may hinder FHRM from satisfying employees’ needs.

Thus, we propose the following hypotheses:

*H3a:* HRM system strength will moderate the relationship between FHRM and the satisfaction of employees’ need for autonomy such that the relationship will be strong (vs. weak) when the HRM system strength is high (vs. low).

*H3b:* HRM system strength will moderate the relationship between FHRM and the satisfaction of employees’ need for competence such that the relationship will be strong (vs. weak) when the HRM system strength is high (vs. low).

*H3c:* HRM system strength will moderate the relationship between FHRM and the satisfaction of employees’ need for relatedness such that the relationship will be strong (vs. weak) when the HRM system strength is high (vs. low).

## Methods

### Sample and procedures

We recruited 1,050 employees and their direct leaders from 105 teams in different industries (e.g., manufacture, service, and IT) in China. In detail, to improve data quality, the survey was anonymous and matched, each team leader and employee should fill out the identification number assigned to them correctly. In addition, to address the common method bias concerns (Podsakoff et al., 2003), we collected the data in two phases. In the first phase, we invited the leaders to rate their FHRM and the employees to provide demographics information (i.e., gender, age, educational background, and work tenure) and report the satisfaction of their three basic psychological needs and their perception of their company’s HRM system strength. The second phase was two weeks after the first phase, when we asked the employees who participated in the first phase to evaluate their workplace well-being.

Specifically, in the first phase, 102 supervisors and 806 employees answered and returned the questionnaire, and in the second phase, 637 employees answered and returned the questionnaire. We obtained a total of 81 paired questionnaires, which consisted of 81 questionnaires from the leaders and 444 questionnaires from the employees. Among the employees in our sample, 56.5% were female and 64% held a college degree. The average age of the employees was 30 years, with most being within the age range of 26–35 years, which accounted for 61.3% of the entire sample.

### Measures

Unless otherwise specified, we used a Likert scale ranging from 1 (“strongly disagree”) to 5 (“strongly agree”) to measure the items.

#### FHRM

We used the 11-item FHRM scale developed by Chang et al. (2013) to measure FHRM. The scale assesses two components, that is, the resource flexibility-oriented HRM subsystem and the coordination flexibility-oriented HRM subsystem, to measure FHRM. Leaders typically have a deep understanding of their company’s HRM strategies, we thus asked the leaders in the sample to rate their corporation’s FHRM. The scale’s Cronbach’s alpha was 0.683, which is slightly below the conventional 0.70 threshold. However, because Cronbach’s alpha tends to underestimate reliability when the tau-equivalent assumption is violated (i.e., when factor loadings vary across items), we additionally computed McDonald’s Omega, which does not require this restrictive assumption (Cortina et al., 2020; Raykov, 1998). Based on the standardized factor loadings from an exploratory factor analysis (forcing a single component), the Omega coefficient was 0.776, exceeding the 0.70 acceptability criterion (Cortina, 1993; McDonald, 1999).

#### HRM system strength

We asked the employees to rate their perceived HRM system strength on a seven-item scale (Hauff et al., 2017). The scale’s Cronbach’s alpha was 0.705.

#### Satisfaction of basic psychological needs

We used 16 items from the basic psychological needs values scale developed by Van den Broeck et al. (2010) to rate satisfaction of basic psychological needs. The scale consists of three components: satisfaction of need for autonomy (6 items), satisfaction of need for competence (4 items), and satisfaction of need for relatedness (6 items). The scale’s Cronbach’s alpha was 0.900.

#### Employees’ workplace well-being

We asked the employees to self-assess their workplace well-being. We used a six-item workplace well-being values scale from the employee well-being scale developed by Zheng et al. (2015) to measure employees’ workplace well-being. The scale’s Cronbach’s alpha was 0.914.

#### Control variables

Prior research indicated that employees’ gender, age, educational background, and tenure within the organization can affect their workplace well-being (Greguras and Diefendorff, 2009). Therefore, we incorporated the factors as control variables in the model. We asked the employees to report their gender (0 = female, 1 = male), age, educational background (1 = high school diploma or lower, 2 = associate’s degree, 3 = bachelor’s degree, 4 = master’s degree), and tenure in their organization.

### Data aggregation

Because employees were nested within teams, we assessed the appropriateness of aggregating individual-level HRM system strength to the team level. We calculated the intra-class correlations (ICCs) and the interrater reliability (rwg). Across all 81 teams (team size range = 3–9 members), the range of rwg values was from 0.774 to 0.983, and the mean rwg(j) was 0.951 (SD = 0.033). In addition, based on 444 individual respondents, the ICC(1) was 0.178 and the ICC(2) was 0.543. Based on these indices, FHRM measures were aggregated to the team level for subsequent analyses.

## Data analysis and results

We examined the discriminant validity by using Mplus 7.0. Considering the substantial number of measurement items in each scale, we conducted random assignment to parcel the items (Little et al., 2002). We classified the items in the FHRM scale under two dimensions (i.e., resource flexibility-oriented HRM and coordination flexibility-oriented HRM). The other scales had a single-dimension structure. We conducted confirmatory factor analysis after parceling the items (Table 1). The six-factor model exhibited good model fit (*χ^2^* [187] = 289.070; CFI = 0.97; SRMR = 0.03; RMSEA = 0.04) and the best fit among the five-factor models.

**Table 1:** Analysis of discriminant validity of predictor variables.

Model	χ^2^	*df*	CFI	TLI	RMSEA	SRMR
Six-factor Model (FHRM, HRMSS, EWW, satisfaction of need for autonomy, competence, and relatedness)	289.070	187	0.97	0.97	0.04	0.03
Five-factor Model A (satisfaction of need for autonomy and competence combined)	388.976	196	0.95	0.94	0.05	0.05
Five-factor Model B (satisfaction of need for autonomy and relatedness combined)	507.895	196	0.92	0.90	0.06	0.05
Five-factor Model C (satisfaction of need for competence and relatedness combined)	318.344	196	0.97	0.96	0.04	0.03
Five-factor Model D (satisfaction of need for autonomy and EWW combined)	1158.653	196	0.74	0.68	0.11	0.11
Five-factor Model E (satisfaction of need for competence and EWW combined)	1037.152	196	0.78	0.72	0.10	0.19
Five-factor Model F (satisfaction of need for relatedness and EWW combined)	1227.556	196	0.73	0.66	0.11	0.11

FHRM = flexible human resource management. EWW = employees’ workplace well-being. HRMSS = HRM system strength. CFI = Comparative Fit Index. TLI = Tucker-Lewis Index. RMSEA = Root Mean Square Error of Approximation. SRMR = Standardized Root Mean Square Residual.

Table 2 presents the zero-order correlations and the descriptive statistics of the variables. The results displayed a positive relationship between FHRM and satisfaction of need for autonomy (*r* = 0.15, *p* < 0.01), satisfaction of need for competence (*r* = 0.20, *p* < 0.01), satisfaction of need for relatedness (*r* = 0.19, *p* < 0.01), and employees’ workplace well-being (*r* = 0.10, *p* < 0.05). Moreover, satisfaction of need for autonomy, competence, and relatedness was positively related to employees’ workplace well-being (*r* = 0.15, *p* < 0.01; *r* = 0.18, *p* < 0.01; *r* = 0.14, *p* < 0.01). The results preliminarily supported our hypotheses and laid the groundwork for further hypothesis testing in the subsequent analyses.

**Table 2:** Means, standard deviations, and correlations among variables.

Variables	M	SD	1	2	3	4	5	6	7	8	9
Individual level											
1. Gender	0.44	0.50									
2. Age	30.21	5.86	0.04								
3. Educational background	2.86	0.72	−0.04	−0.01							
4. Tenure in organization	35.29	25.97	0.06	0.40^**^	0.01						
5. Satisfaction of need for autonomy	3.68	0.81	0.02	0.08	−0.02	0.15^**^					
6. Satisfaction of need for competence	4.18	0.57	0.02	0.17^**^	−0.03	0.22^**^	0.66^**^				
7. Satisfaction of need for relatedness	4.18	0.58	0.08	0.17^**^	0.02	0.20^**^	0.59^**^	0.66^**^			
8. Employees’ workplace well-being	4.05	0.61	−0.06	−0.04	0.09	−0.03	0.15^**^	0.18^**^	0.14^**^		
Organization level											
9. FHRM	4.25	0.37	0.08	0.04	−0.07	−0.00	0.15^**^	0.20^**^	0.19^**^	0.10^*^	
10. HRM system strength	4.09	0.25	−0.03	0.03	0.04	0.05	0.31^**^	0.31^**^	0.31^**^	0.08	0.34^**^

*N* = 444. FHRM = human resource management. M = mean. SD = standard error. ***p<0.001, **p<0.01, *p<0.05.

To test our hypotheses, two-level path analysis was conducted in Mplus 7.0 with MLR estimation, with FHRM and HRM system strength specified as a between-team level and all other variables (three psychological needs, workplace well-being, and control variables: gender, age, education, and tenure) specified at the within-individual level. In Hypothesis 1, we proposed that FHRM can positively affect employees’ workplace well-being. The regression results are presented in Table 3, which shows that FHRM can positively predict employees’ workplace well-being (*β* = 0.19; *p* < 0.01). Thus, Hypothesis 1 was supported.

**Table 3:** Results of path analysis: mediating effect.

Path	Estimates	SE	95% CI
Main effect			
FHRM → workplace well-being	0.19^**^	0.07	[0.052, 0.328]
Direct effect (controlling for each mediator)			
FHRM → workplace well-being (controlling for satisfaction of need for autonomy)	0.15^*^	0.07	[0.019, 0.286]
FHRM → workplace well-being (controlling for satisfaction of need for competence)	0.13	0.07	[−0.003, 0.262]
FHRM → workplace well-being (controlling for satisfaction of need for relatedness)	0.15*	0.07	[0.013, 0.281]
Mediating effect			
FHRM → satisfaction of need for autonomy → workplace well-being	0.04^*^	0.02	[0.001, 0.073]
FHRM → satisfaction of need for competence → workplace well-being	0.06^**^	0.02	[0.020, 0.099]
FHRM → satisfaction of need for relatedness → workplace well-being	0.04^*^	0.02	[0.006, 0.079]

*N* = 444. SE = standard error. CI = Confidence Interval. ***p<0.001, **p<0.01, *p<0.05.

Following this, to test the mediation effects, we estimated three separate single-mediator models, given the moderate correlations among the three mediators (*r* = 0.59–0.66) to avoid estimation instability (Preacher and Hayes, 2008). The 95%CI for indirect effects were obtained via the Delta method. As shown in Table 3, all three indirect effects were significant, supporting Hypotheses 2a, 2c. The indirect effects were modest in magnitude, suggesting that each need satisfaction explains a small but significant portion of the total effect. Specifically, satisfaction of need for autonomy (*β* = 0.04, *p* < 0.05, 95% CI [0.001, 0.073]) and relatedness (*β* = 0.04, *p* < 0.05, 95% CI [0.006, 0.079]) partially mediated the FHRM–well-being relationship, as their direct effects remained significant (autonomy: *β* = 0.15, *p* < 0.05, 95% CI [0.019, 0.286]; relatedness: *β* = 0.15, *p* < 0.05, 95% CI [0.013, 0.281]), with both 95% CIs excluding zero. In contrast, satisfaction of need for competence fully mediated this relationship (indirect effect: *β* = 0.06, *p* < 0.01, 95% CI [0.020, 0.099]; direct effect: *β* = 0.13, *p* = 0.056, 95% CI [−0.003, 0.262]), as the direct effect became non-significant after controlling for competence, with its 95% CI containing zero.

**Figure 2:**
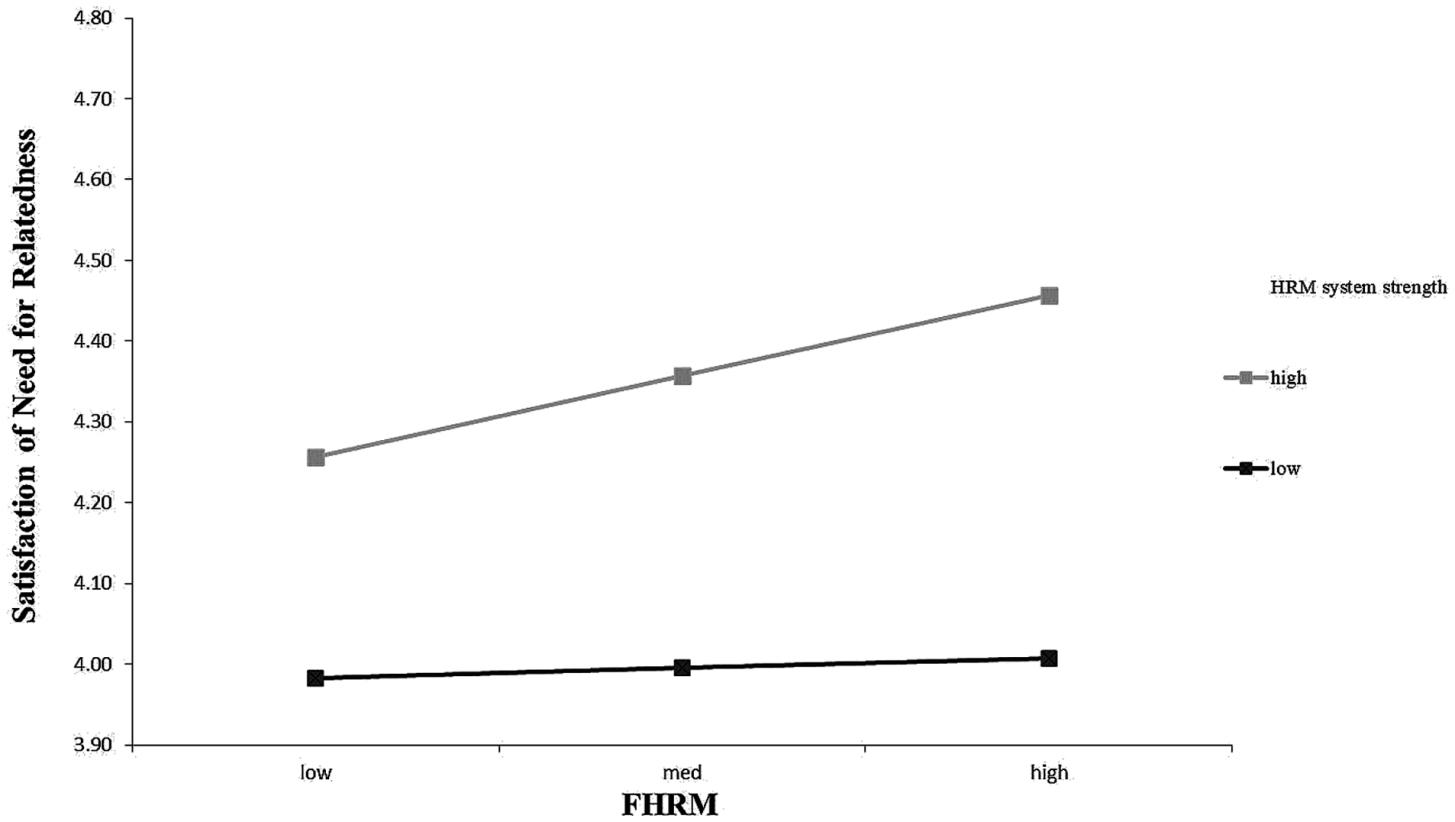
Moderating effect of HRM system strength on the relationship between FHRM and satisfaction of need for relatedness.

FHRM and HRM system strength were grand-mean centered before creating the interaction term. The results (Table 4) showed that the simple slopes of FHRM on autonomy need satisfaction were not significant at either high (+1 SD) or low (−1 SD) levels of HRM system strength (*β*_+1SD = 0.16, *p* > 0.05; *β*_−1SD = 0.06, *p* > 0.05). Hence, hypothesis 3a was not supported. Similarly, HRM system strength also did not moderate the effect of FHRM on satisfaction of basic psychological need for competence, because the simple slope of FHRM was significant at high (+1 SD) levels of HRM system strength (*β*_+1SD = 0.19, *p* < 0.01), it was not significant at low (−1 SD) levels (*β*_−1SD = 0.15, *p* > 0.05), and the difference between the two slopes was non-significant (*β*_diff = 0.05, *p* > 0.05). Therefore, hypothesis 3b was not supported. For hypothesis 3c, the results of the regression analysis indicated that the interaction term (FHRM × HRM system strength) significantly predicted the satisfaction of need for relatedness (*β* = 0.41, *p* < 0.05), and its inclusion explained an additional 0.6% of the variance beyond the main effects (ΔR^2^ = 0.006). Although the incremental variance explained by the interaction was modest, the significant interaction coefficient, which was moderate in effect size, and the clear slope pattern support the moderating role of HRM system strength. Accordingly, as illustrated in Figure 2, the simple slope test results showed that the moderating effect was significant at high (+1 SD) levels of HRM system strength (*β_*+1SD = 0.24, *p* < 0.001) but not significant at low (−1 SD) levels (*β*_−1SD = 0.04, *p* > 0.05). In addition, the difference between the high and low levels was significant (*β*_diff = 0.20, *p* < 0.05). Thus, hypothesis 3c was supported.

**Table 4:** Results of path analysis: moderating effect.

Path	Estimates		SE
Moderating effect			
FHRM × HRM system strength → satisfaction of need for autonomy	+1 SD	0.16	0.12
	−1 SD	0.06	0.10
	Difference	0.09	0.12
FHRM × HRM system strength → satisfaction of need for competence	+1 SD	0.19^**^	0.07
	−1 SD	0.15	0.09
	Difference	0.05	0.11
FHRM × HRM system strength → satisfaction of need for relatedness	+1 SD	0.24^***^	0.07
	−1 SD	0.04	0.08
	Difference	0.20^*^	0.10

*N* = 444. FHRM = flexible human resource management. SE = standard error. ***p<0.001, **p<0.01, *p<0.05.

In conclusion, our results confirm that FHRM can positively influence employees’ workplace well-being, and satisfaction of basic psychological needs (i.e., autonomy, competence, and relatedness) can mediate the relationship between FHRM and employees’ workplace well-being. Moreover, HRM system strength can moderate the relationship between FHRM and satisfaction of need for relatedness. Furthermore, in organizations with a strong HRM system, a high level of FHRM can effectively satisfy employees’ need for relatedness and thus enhance their workplace well-being.

## Discussion

### Discussion of findings

Our study examined the mechanisms and boundary conditions underlying the relationship between FHRM and workplace well-being. Our results show that FHRM positively predicts workplace well-being. This finding is consistent with prior evidence demonstrating that flexibility-related practices in isolation, such as flexible job design or work arrangements, are conducive to individual well-being (Diep and Horváthová, 2026; Fox et al., 2022), and also provides empirical support for this argument at the level of integrated HRM practices. In addition, our results indicate that satisfaction of the three psychological needs (autonomy, competence, and relatedness) mediates the effect of FHRM on well-being, which is consistent with Wu et al. (2024), who found that psychological need satisfaction promotes employee well-being.

When aggregating HRM system strength to the team level, the ICC(2) value (0.543) was moderate, which is partly attributable to the relatively small team sizes in our sample (M = 5.5 employees per team), as ICC(2) is sensitive to group size (Bliese, 2000). Meanwhile, the ICC(1) value (0.178) falls within the typical range for field studies and can be considered a moderate to large effect size, which supports the appropriateness to the team level (James, 1982; LeBreton and Senter, 2008). Taken together, these indices support the appropriateness of aggregating HRM system strength to the team level. Furthermore, our analytical approach is similar to that of Sanders et al. (2008) in that we aggregated HRM system strength to the organizational level and treated it as a cross-level moderator. Moreover, our findings regarding the moderating role of HRM system strength are consistent with those of Sanders et al. (2008), providing further evidence that HRM system strength amplifies the effects of HRM practices on individual employee outcomes.

Notably, although the moderation results show that the effects of FHRM on the satisfaction of need for autonomy and competence become non-significant under high HRM system strength, this can be explained by strong HRM systems amplifying multiple, sometimes competing, signals, that is, beyond FHRM, other practices may counteract its intended effects (Snell and Wright, 1998). For example, contract type and overtime agreements can constrain autonomy, while flexible staffing may hinder competency development, as building competencies takes time and a long-term view (Hauff et al., 2017; Osterman, 1987). In contrast, the satisfaction of need for relatedness was positively moderated by HRM system strength. A plausible explanation is that FHRM practices, including participative management, information communication and fostering collaboration, do not conflict with other organizational HRM aspects in terms of their relational signals. When HRM system strength is high, these relational signals are transmitted clearly and consistently to all employees, who then develop shared perceptions and act cooperatively, thereby fulfilling their need for relatedness. Another explanation concerns the differential nature of these needs: unlike autonomy and competence, which rely on internal cognitive appraisals (assessing whether they have control or are growing skills), relatedness satisfaction is largely driven by observable and concrete behavioral interactions, such as information sharing and participative decision-making (Deci and Ryan, 2000). Thus, when HRM system strength is high, these explicit relational cues are encoded clearly and acted upon consistently, making the moderating effect on relatedness more robust.

### Theoretical implications

Our research makes several contributions to the literature on FHRM. First, a central contribution of this study is to establish workplace well-being as a meaningful consequence of FHRM, thereby shifting the dominant research focus from organizational-level and behavioral outcomes toward the individual psychological states (e.g., Bal and De Lange, 2015; Ben Guedria et al., 2025; Chang et al., 2013; Lakshman et al., 2022). By demonstrating that FHRM positively predicts well-being, our findings not only extend the nomological network of FHRM but also answer calls for HRM research to move beyond the “performance-only” view and to treat employee well-being not as a secondary concern but as a legitimate and measurable outcome of strategic HRM systems (Guest, 2017; Van De Voorde et al., 2012). This theoretical extension broadens the evaluative criteria for FHRM effectiveness and enriches FHRM theorizing.

Second, our study expands theoretical perspectives on the mechanisms of FHRM. Previous studies have investigated the impact of FHRM primarily through two theoretical lenses: a resource-based perspective (Bal and De Lange, 2015; Zhang et al., 2023) and a capability-based perspective (Chang et al., 2013; Islam et al., 2025; Li and Lin, 2024). By identifying the satisfaction of basic psychological needs (autonomy, competence, and relatedness) as a mediating pathway, our study provides intrinsic psychological conditions to explain how FHRM translates into workplace well-being. Additionally, this account based on self-determination theory provides a more foundational explanation for FHRM effects by revealing that flexible HRM practices are not merely strategic tools for organizational adaptation, but also proximal determinants of individual intrinsic motivation and psychological well-being.

Third, our study uses HRM system strength as a moderator and integrates two major perspectives, namely, “content” and “processes,” into HRM. Prior research has largely focused on what FHRM practices are and what outcomes they produce (content), with individual-level studies placing greater emphasis on boundary conditions such as leadership or individual traits (e.g., Xu et al., 2023; Zhang et al., 2023), yet limited attention has been paid to whether these HRM practices are communicated with clarity and consistency and perceived distinctively and consensually by employees (process). Thus, the result of HRM system strength serving as a moderating role reveals a critical theoretical boundary: the effectiveness of FHRM depends not only on the design of its practices but also on the strength of the system that communicates them. By integrating these two perspectives, our study offers a more comprehensive understanding of FHRM effects and highlights the importance of considering both the content and the signaling quality of HRM systems.

### Practical implications

Our research has practical implications for organizations. First, organizations should expedite the development of FHRM systems through strategic resource acquisition and allocation. Our findings suggest that FHRM can meet employees’ basic psychological needs and improve their workplace well-being. Moreover, an increase in employees’ workplace well-being can improve their attitude toward work and behavior. Our results highlight the need for organizations to prioritize the design and optimization of HRM systems, which will enable them to implement FHRM, adapt to the dynamic environment, and align their goals of employee well-being enhancement and organizational development. Specifically, organizations can help their employees broaden their knowledge and hone their skills by fostering resource flexibility while implementing adaptive management to achieve alignment in dynamic competitive environments through coordination flexibility.

Second, organizations and managers should jointly satisfy employees’ need for autonomy, competence, and relatedness from various aspects. As living standards improve, new generations of employees will increasingly seek positive emotions and self-actualization from work, instead of focusing on material satisfaction. Satisfaction of employees’ need for autonomy, competence, and relatedness can serve as a key mediator in the relationship between FHRM and employees’ workplace well-being. Therefore, organizations and leaders should attach considerable importance to the satisfaction of employees’ basic psychological needs as a basic management practice. Specifically, organizations can encourage their employees to participate in decision making and actively provide suggestions to meet their need for autonomy. Moreover, leaders should offer guidance and support to employees to meet their need for competence. Meanwhile, to satisfy employees’ need for relatedness, organizations should foster the creation of a positive climate, reinforce employees’ psychological contracts and sense of belonging, and demonstrate care, support, and trust toward their employees.

Third, organizations and managers must focus on HRM content and implementation processes to maximize its utility. Our results show that the higher the HRM system strength, the stronger the direct effect of FHRM on satisfaction of the need for relatedness. This finding suggests that organizations should ensure that their employees understand and identify with their relevant HRM policies. Organizations should tailor HRM policies to specific needs and promptly communicate their policies to their employees. Moreover, organizations should establish feedback channels to help their employees understand the issues that need to be resolved, as well as the expected goals of the policies. Furthermore, managers should reach a consensus on the policies, ensure fairness in the implementation process, and promote the universal recognition of organizational HRM among the employees.

### Limitations and future directions

Our research has several limitations that can serve as research avenues for future works. First, we used a time-lagged model and collected our data from leaders and employees in two phases. Our design effectively addressed the common method bias concerns. However, because we collected data on the antecedent variable (FHRM reported by the leaders) and the mediator variable (satisfaction of basic psychological needs reported by the employees) in the same phase, we established a causal relationship between FHRM, satisfaction of basic psychological needs, and employees’ workplace well-being. FHRM implementation will have a delayed effect on employees’ workplace well-being; thus, future research can adopt a longitudinal design to accurately investigate and establish the causal relationship between the variables. Additionally, while we used multi-source data for key variables, some constructs (e.g., basic psychological needs satisfaction and well-being) were measured via self-reports, which may introduce self-report biases that could inflate the observed relationships. Future studies could incorporate objective indicators or other-rated assessments to complement self-reported measures and further mitigate CMB concerns.

Second, our conclusions can enrich research on the relationship between FHRM and individual-level variables. However, employees’ well-being can manifest in various forms. In addition, HRM systems can influence other aspects of employees’ well-being (Zhang et al., 2022). Future research can use the employee workplace well-being scale developed by Zheng et al. (2015) to explore the impact of FHRM on employees’ general well-being and psychological well-being across various dimensions.

Third, we used HRM system strength to integrate the two perspectives of HRM “content” and “processes.” Organizations’ FHRM implementation may be affected by other contextual factors related to the satisfaction of employees’ basic psychological needs. Future research can investigate the impact of other moderators to effectively understand the boundary conditions of the effectiveness of FHRM.

Fourth, we employed three independent single-mediator models instead of an integrated parallel mediation model. As Preacher and Hayes (2008) demonstrated, high correlations between mediators cause collinearity suppression in joint models, reducing the size of b-paths and yielding non-significant indirect effects in our full parallel model. Separate regressions identify each mediator’s unique indirect role but cannot compare the relative strength of different indirect mechanisms simultaneously. Future research can improve scale design or expand sample size to decrease mediator inter-correlation and conduct robust parallel mediation analysis.

## Data Availability

The raw data supporting the conclusions of this article will be made available by the authors, without undue reservation.
